# Evaluation of the Relationship Between the Grade of Lumbar Foraminal Spinal Stenosis and Outcomes of Dorsal Root Ganglion Pulsed Radiofrequency Treatment

**DOI:** 10.1155/prm/2303107

**Published:** 2026-01-29

**Authors:** Hamit Göksu, Mehmet Çetin Başkaya

**Affiliations:** ^1^ Department of Pain, Ankara Dr. Abdurrahman Yurtaslan Oncology Training and Research Hospital, Ankara, Turkey

**Keywords:** low back pain, patient outcome assessment, pulsed radiofrequency treatment, spinal stenosis

## Abstract

**Objective:**

We aimed to evaluate the association between the radiological grade of lumbar foraminal spinal stenosis (LFSS) and the outcomes of dorsal root ganglion (DRG) pulsed radiofrequency (PRF) treatment.

**Materials and Methods:**

This is an observational, single‐center study. Patients with LFSS who had undergone lumbar DRG‐PRF treatment were evaluated according to the radiological grade of stenosis: Grades 1, 2, and 3. Severity of pain, presence of neuropathic pain, and functional status were assessed using a numerical rating scale (NRS), the Douleur Neuropathique en 4 (DN4) Questionnaire, and the Oswestry Disability Index (ODI) at baseline, first, and third months. The groups by grade consisted of 18, 22, and 23 patients, respectively, for Grades 1, 2, and 3.

**Results:**

NRS scores are similar at baseline and first month, but higher in Group 3 than in Groups 1 and 2 at the third month (*p* = 0.010, *p* = 0.04). Similarly, DN4 scores are similar at baseline and first month, but higher in Group 3 than in Group 1 (*p* = 0.017). ODI scores and weekly analgesic intake at baseline, first, and third months are similar. There are significant decreases in the NRS, DN4, ODI, and weekly analgesic consumption in all groups during follow‐up (*p* < 0.05). The ratios of meaningful pain relief were 72.2%, 68.2%, and 69.6% at the first month, and 50.0%, 63.6%, and 43.5% at the third month for Grades 1, 2, and 3 groups, respectively, without significant differences at the first and third months (*p* > 0.05).

**Conclusion:**

The DRG‐PRF treatment is effective for pain and functional disability in LFSS in all grades, although pain scores remained higher in Grade 3 stenosis at the third month. Studies with larger sample sizes for each stenosis grade may provide more accurate and detailed information.

## 1. Introduction

**Figure 1:**
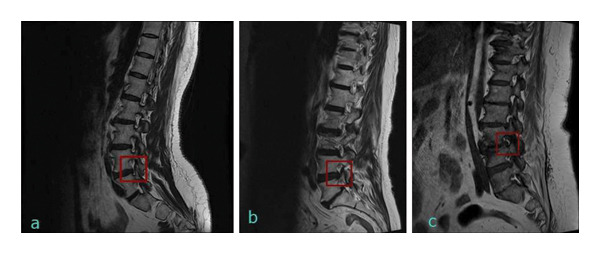
Grade 1 (a), Grade 2 (b), and Grade 3 (c) foraminal spinal stenosis.

Lumbar foraminal spinal stenosis (LFSS) is defined as the narrowing of the bony exit of the nerve root due to the decrease in the height of the intervertebral disc, osteoarthritic changes of the facet joints, cephalad subluxation of the superior articular process of the inferior vertebra, and sprain/torsion of the ligamentum flavum or protrusion of the annulus fibrosus [1]. Acquired stenosis develops secondary to degenerative changes in the spine, including facet joint degeneration, ligamentous laxity, bone hypertrophy, disc disorders, and osteophyte formation [2]. Patients with foraminal neuropathy present with radicular symptoms and/or neurogenic claudication. Magnetic resonance imaging (MRI) can identify nerve compression, determine the cross‐sectional area of the spinal canal, and assess the severity of foraminal stenosis. Foraminal stenosis is graded according to perineural fat obliteration or foraminal size. Grade 1 refers to a mild degree of foraminal stenosis, showing perineural fat obliteration surrounding the nerve root in the transverse or vertical direction. Grade 2 refers to moderate stenosis in the vertical and transverse directions. There is a narrowing of the foraminal width and height, but no evidence of morphologic changes in the nerve root. Grade 3 represents an advanced degree characterized by collapse or morphologic changes in the nerve root [1] (Figure 1).

Rest, physical therapy modalities, nonsteroidal anti‐inflammatory agents, and agents such as tramadol, oxycodone, morphine, and gabapentinoids can be used to treat lumbar foraminal stenosis [3]. Interlaminar or transforaminal epidural injections [4], percutaneous adhesiolysis [5], percutaneous neuroplasty [6], epiduroscopy, balloon adhesiolysis [7], pulsed radiofrequency (PRF) to the dorsal root ganglion (DRG) [8], electrical stimulation to the DRG [9], and spinal cord stimulation [10] can be used in cases resistant to conservative treatment. Surgery is the final option for patients unresponsive to these interventional pain treatments.

DRG‐PRF neurostimulation has emerged, as it has proven to be effective in the control of various chronic pain problems, particularly for the relief of neuropathic pain [11]. DRG‐PRF therapy is one of the first recommended treatment options, along with epidural injections for pain refractory to conservative treatments [12]. DRG‐PRF is a potentially attractive alternative to epidural steroid injection in the treatment of chronic radicular pain. It is target‐specific, can be administered to multiple roots, and avoids the side effects of steroid use for patients at risk of receiving these agents [13]. Although PRF to the lumbar DRG is one of the most common interventional pain treatments for lumbar foraminal stenosis, we found no studies evaluating the association between the degree of foraminal stenosis and the efficacy of PRF treatment. In a previous study comparing the effectiveness of transforaminal epidural injections between the two groups with mild to moderate and severe foraminal stenosis, the efficacy of transforaminal epidural injections was found to be better in the group with mild to moderate stenosis [13]. In the current study, we investigated the relationship between the degree of LFSS and the outcomes of dorsal root PRF treatment.

## 2. Materials and Methods

### 2.1. Study Design and Patients

**Figure 2:**
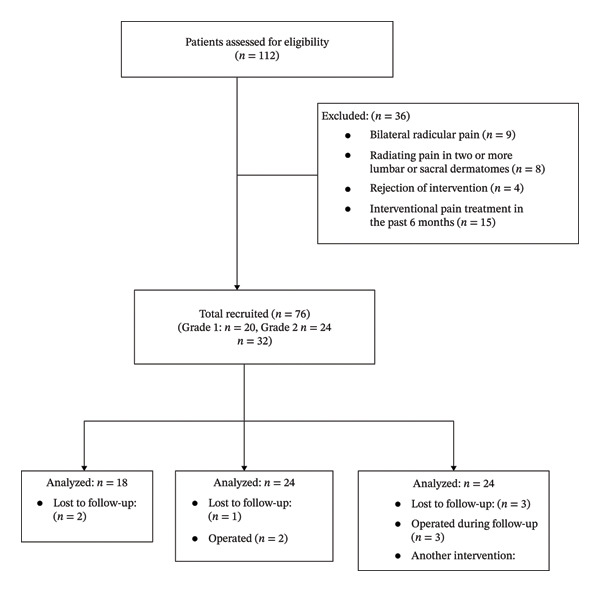
The flowchart of the study.

This study was conducted between January 1, 2024, and August 31, 2024, in the pain medicine department of our hospital. Ethical committee approval was obtained for the study (Number: 2023‐07/285). The study was conducted in accordance with the principles outlined in the Declaration of Helsinki. Inclusion criteria were as follows: 20–75 years of age, radicular low back pain, foraminal stenosis secondary to foraminal disc pathology or degenerative changes detected by MRI, no additional pathology on imaging to cause pain, persistent pain for at least 3 months despite medical and conservative treatment methods, a pain severity of 5 or more on the 0–10 numeric rating scale, and acceptance and signature of informed written consent form. The radiological grade of stenosis was determined by a radiologist with 15 years of experience in MRI. Patients with bilateral radicular pain or symptoms of multiple spinal levels; a history of fracture, malignancy, bone marrow disease, cancer, drug, and opiate addiction; previous spinal surgery; patients with symptoms such as weight loss, fever, night sweats, and nocturnal pain; patients with motor deficits; patients with infection or elevated acute phase protein in laboratory findings; patients who have undergone interventional pain treatment in the past 6 months; patients for whom interventions were not completed for any reason; and patients with deficient data or missing any of the follow‐ups at first and third months were excluded from the study. The study was completed with 63 patients: 18 with Grade 1, 22 with Grade 2, and 23 with Grade 3 foraminal stenosis. The flowchart of the study is presented in Figure 2.

### 2.2. Interventions and Measurements

**Figure 3:**
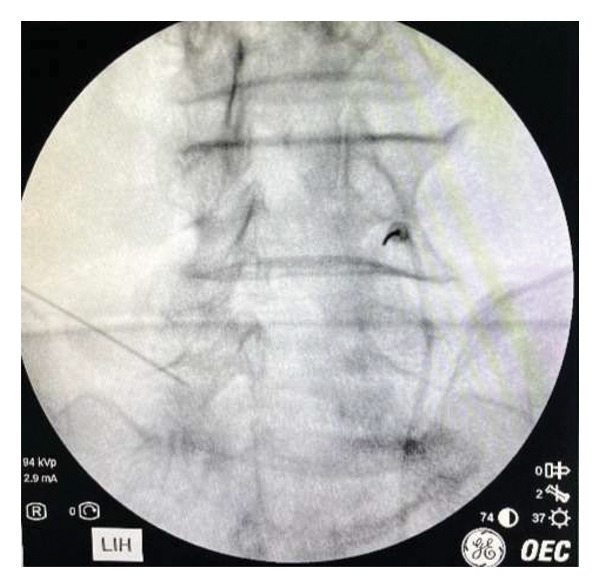
The lumbar dorsal root ganglion pulsed radiofrequency treatment.

The DRG‐PRF procedure was performed using PRF at 45 V and 42° for 4 min to the DRG, utilizing sensory and motor stimulation with 100 mm long, 10‐mm active‐tipped temperature‐controlled needles, under the guidance of fluoroscopy from the disc level where the patient had symptomatic foraminal stenosis and consistent with clinical findings (Figure 3). The interventions were performed without sedation, as in our routine practice. After the procedure, the patients were continuously monitored and discharged 1 h later if no complications were observed. Any side effects and complications were recorded. The demographic, clinical features, and outcome measurements were compared between groups assigned by grade of foraminal stenosis, categorized as Grades 1, 2, and 3.

The patients′ radicular pain scores at admission and first and third months were evaluated using a numerical rating scale (NRS), and the presence of neuropathic pain was assessed using the Douleur Neuropathique en 4 Questionnaire (DN4) [14]. The functional status was assessed using the Oswestry Disability Index (ODI). ≥ 50% Pain relief, as indicated by NRS scores, was considered a meaningful pain relief. The number of analgesics consumed per week was recorded. Reliability and validity analyses of the DN4 and ODI were conducted in our country [15, 16]. The number of analgesics consumed per week, including paracetamol, nonsteroidal anti‐inflammatory drugs, tramadol, or tramadol/paracetamol combinations, by patients before and after the procedure, was recorded. The primary outcome was meaningful pain relief, and the secondary outcomes were improvements in NRS, DN4, and ODI scores. The outcome scores were assessed by another pain specialist who did not perform interventions.

### 2.3. Sample Size Calculation and Statistical Analysis

Power analysis was performed using G^∗^Power Version 3.1.9.4. By comparing the meaningful pain reduction rates (87.1% and 42.3%) of patients with mild–moderate and severe stenosis in the third month after transforaminal epidural injection, as reported by Chang and Lee [13], it was determined that a total of 50 patients should be included in the study, with a margin of error of 0.05% and 95% power.

Recorded data were analyzed using the Statistical Package for Social Sciences, Version 27.0 (SPSS Inc., Armonk, NY). The normality of the numerical data distribution was examined using the Shapiro–Wilk normality test. Normally distributed continuous variables were presented as mean ± standard deviation (SD), while non‐normally distributed ones were presented with median and interquartile range (IQR) (25th–75th percentiles). Qualitative data were expressed as frequencies and percentages. The Pearson chi‐square, Fisher’s exact test, and likelihood ratio were used to compare categorical variables. Paired samples *t*‐test and Wilcoxon signed–rank tests were used to analyze repeated numeric variables according to a parametric distribution. The Kruskal–Wallis test, followed by post hoc Bonferroni tests, was performed to compare more than two groups. An ANCOVA analysis was performed on ODI scores, with age as a covariate, after verifying the assumption of homogeneity of regression slopes to compare the groups. The confidence interval was set at 95%, and the accepted margin of error was 5%; thus, *p* < 0.05 was considered significant.

## 3. Results

The demographic and clinical features of the groups assigned by grade of foraminal stenosis are presented in Table 1. The age of the patients is significantly lower in Group 1 than in Groups 2 and 3 (*p* < 0.01); no significant difference is found between Groups 2 and 3 (*p* = 0.685). There are no significant differences in gender, BMI, or smoking status between groups (*p* > 0.05). Occupation and stenosis level could not be analyzed due to the limited number of cases. The duration and side of pain do not differ significantly between the groups (*p* > 0.05). Only two patients in the Grade 1 group experienced hypotension after the completion of the intervention, which was treated with serum physiologic infusion and did not necessitate any medications. No complications were observed that would hinder treatment in any patient.

**Table 1:** The demographic and clinical data of the patient groups.

Variables	Grade 1 (*n* = 18)	Grade 2 (*n* = 22)	Grade 3 (*n* = 23)	*p* value
Age (mean ± SD)	53.2 ± 10.8	64.3 ± 9.1	66.9 ± 11.4	**<** **0.001** ^∗^
Gender (*n*/%)				0.728
Female	13 (72.2)	17 (77.3)	19 (82.6)	
Male	5 (27.8)	5 (22.7)	4 (17.4)	
BMI (mean ± SD)	26.6 ± 3.1	28.2 ± 3.3	29.3 ± 3.5	0.058^∗^
Occupation (*n*/%)				
Housewife	9 (50.0)	15 (68.2)	16 (69.6)	
Retired	5 (27.8)	6 (27.3)	4 (17.4)	
Worker	3 (16.7)	—	2 (8.7)	
Officer	1 (5.6)	1 (4.5)	1 (4.3)	
Smoking (*n*/%)	5 (50.0)	15 (68.2)	16 (69.6)	0.370
Pain duration (months) (median; IQR)	10.5 (38.5)	8.5 (6.8)	7.0 (15.0)	0.091
Side of pain (*n*/%)				0.527
Right	6 (33.3)	11 (50.0)	11 (47.8)	
Left	12 (66.7)	11 (50.0)	14 (52.2)	
Level of symptomatic foraminal stenosis (*n*/%)				
L2‐3	—	1 (4.5)	1 (4.3)	
L3‐4	—	2 (9.1)	3 (13.0)	
L4‐5	14 (77.8)	11 (50.0)	12 (52.2)	
L5‐S1	4 (22.2)	8 (36.4)	7 (30.4)	

*Note:* The bold values indicate significant *p*‐values.

Abbreviations: CAD, coronary artery disease; DM, diabetes mellitus; HT, hypertension; IQR, interquartile range; SD, standard deviation.

^∗^One‐way ANOVA analysis.

^†^Fisher’s exact test.

### 3.1. Disease

**Figure 4:**
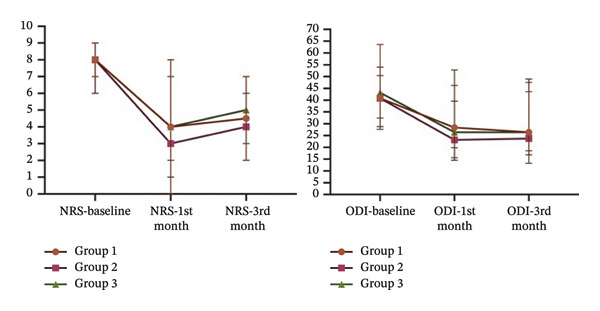
Changes of the numeric rating scale and Oswestry Disability Index scores of the groups.

NRS scores are similar at baseline and first month, but higher in Group 3 than in Groups 1 and 2 at the third‐month follow‐up (*p* = 0.010, *p* = 0.04). Similarly, DN4 scores are similar at baseline and first month, but higher in Group 3 than in Group 1 at third‐month follow‐up (*p* = 0.017); no significant differences are found in the other pairwise comparisons (*p* > 0.05). ODI scores and weekly analgesic intake at baseline, first, and third months are similar between the groups (*p* > 0.05). There are significant decreases in the NRS, DN4, ODI, and weekly analgesic consumption in all groups at first and third months compared to baseline in intragroup comparisons (*p* < 0.05). Intra‐ and intergroup comparisons of NRS, DN4, ODI, and the number of analgesics per week are shown in Table 2. The changes in NRS and ODI scores during follow‐up are demonstrated in Figure 4.

**Table 2:** The comparison of outcome measurements during follow‐up.

Variables	Grade 1 (*n* = 18)	Grade 2 (*n* = 22)	Grade 3 (*n* = 23)	*p* value
Mean ± standard deviation or median (interquartile range)				
*NRS scores*				
Baseline	8.0 (7.0–8.3)	7.5 (6.8–8.3)	8.0 (7.0–9.0)	0.349^‡^
1^st^ Month	3.0 (3.0–5.0)	3.0 (3.0–6.0)	4.0 (3.0–5.0)	0.645^‡^
*p* ^∗^	**<** **0.001**	**<** **0.001**	**<** **0.001**	
3^rd^ Month	4.0 (3.0–6.0)	4.0 (3.0–4.0)	5.0 (4.0–6.0)	**0.015** ^‡^
*p* ^∗^	**<** **0.001**	**<** **0.001**	**<** **0.001**	

*DN4 scores*				
Baseline	6.0 (4.0–7.0)	6.0 (4.0–7.0)	6.0 (4.0–7.0)	0.935^‡^
1^st^ Month	3.0 (2.8–4.0)	3.0 (2.0–4.3)	3.0 (2.0–4.0)	0.507^‡^
*p* ^∗^	**0.014**	**<** **0.01**	**<** **0.001**	
3^rd^ Month	3.0 (3.0–4.5)	3.0 (2.0–4.0)	3.0 (2.0–3.0)	**0.021** ^‡^
*p* ^∗^	**0.018**	**<** **0.001**	**<** **0.001**	

*ODI scores*				
Baseline	42.7 ± 8.3	40.3 ± 8.4	41.5 ± 6.1	0.511^§^
1^st^ Month	29.4 ± 7.8	25.0 ± 6.7	28.7 ± 9.4	0.110^§^
*p* ^†^	**<** **0.001**	**<** **0.001**	**<** **0.001**	
3^rd^ Month	29.5 ± 8.0	25.4 ± 7.2	26.6 ± 9.1	0.122^§^
*p* ^†^	**<** **0.001**	**<** **0.001**	**<** **0.001**	

*Number of analgesics per week*				
Baseline	7.0 (6.3–11.0)	7.0 (4.8–12.5)	9.0 (7.0–14.0)	0.358^‡^
1^st^ Month	3.0 (2.8–7.0)	2.5 (2.0–5.0)	4.0 (3.0–7.0)	0.252^‡^
*p* ^∗^	**<** **0.001**	**<** **0.001**	**<** **0.001**	
3^rd^ Month	3.0 (2.0–4.8)	2.5 (2.0–3.3)	3.0 (2.0–5.09)	0.353^‡^
*p* ^∗^	**<** **0.001**	**<** **0.001**	**<** **0.001**	

*Note:* The *p* values in each row were obtained from the comparison of the follow‐up time scores in the upper row versus the baseline scores. The *p* values in each column represent the results of intergroup comparisons at baseline and during follow‐up. DN4, Douleur Neuropathique en 4 Questionnaire. Bold values indicate significant *p*‐values.

Abbreviations: NRS, numeric rating scale; ODI, Oswestry Disability Index.

^∗^Wilcoxon signed–rank test.

^†^Paired samples *t*‐test.

^‡^Kruskal–Wallis test with Bonferroni correction.

^§^ANCOVA analysis using the post hoc Bonferroni test.

No statistically significant differences were found in the ratio of meaningful pain relief between the first and third months, or in changes in NRS and OD scores between the groups (*p* > 0.05) (Table 3).

**Table 3:** The comparison of ratios of meaningful pain relief and changes in numeric rating scale and Oswestry Disability Index scores between the groups.

Variables	Grade 1 (*n* = 18)	Grade 2 (*n* = 22)	Grade 3 (*n* = 23)	*p* value
*Meaningful pain relief (≥* *%50 decrease in NRS score)*				
1^st^ Month	13 (72.2)	15 (68.2)	16 (69.6)	0.962^∗^
3^rd^ Month	9 (50.0)	14 (63.6)	14 (43.5)	0.389^∗^

*Changes in NRS and ODI scores*				
NRS‐baseline/NRS‐1^st^ month	4.2 ± 1.8	3.76 ± 1.2	3.8 ± 1.9	0.668^†^
NRS‐baseline/NRS‐3^rd^ month	3.5 ± 0.8	3.9 ± 1.6	3.2 ± 1.4	0.235^†^
ODI‐baseline/ODI‐1^st^ month	13.3 ± 6.4	15.3 ± 7.3	12.8 ± 6.0	0.627^†^
ODI‐baseline/ODI‐1^st^ month	13.4 ± 5.9	14.9 ± 6.5	14.9 ± 8.7	0.826^†^

Abbreviations: NRS, numeric rating scale; ODI, Oswestry Disability Index.

^∗^Pearson chi‐square test.

^†^One‐way ANOVA analysis.

## 4. Discussion

In the current study, we investigated the relationship between the results of DRG‐PRF treatment and the radiologic stenosis grade. The lower age of the patient group with Grade 1 stenosis is expected, as foraminal stenosis typically worsens with age. Although there were no significant differences in meaningful pain relief ratios, ODI scores, or changes in NRS and ODI scores during follow‐up, we found that NRS scores were higher in Group 3 than in Groups 1 and 2, and the DN4 score was higher in Group 3 than in Group 1. In their study comparing the effect of transforaminal steroid injection in patients with Grades 1‐2 and Grade 3 stenosis as two groups, Chang and Lee [13] found that the decrease in NRS scores at 1, 2, and 3 months after transforaminal epidural steroid injection was higher in those with mild to moderate stenosis, and the meaningful pain relief at the third month was higher in the patients with mild to moderate stenosis. In this study, the small number of patients with Grade 1 stenosis may confound the results. However, we would like to compare all stenosis grades against one another in LFSS.

DRG‐PRF is a potentially compelling alternative to epidural steroid injection for the treatment of chronic radicular pain. It is target‐specific, can be administered to multiple roots, and avoids the side effects of steroid use for patients at low risk of receiving these agents. It has been demonstrated to have both immunomodulatory and neuromodulatory effects. Many studies have reported that DRG‐PRF is an effective treatment modality for lumbosacral radicular pain [17–20]. However, we did not find any studies that evaluated the outcomes of DRG‐PRF with respect to LFSS grade. Therefore, some studies showed little evidence of DRG‐PRF. A review by Park et al. [21] reported that DRG‐PRF showed no differences in pain scores at first and sixth months. They also noted no significant difference in ODI scores at the first, third, and sixth months. However, they reported that there is low‐quality evidence that DRG‐PRF application had a more significant analgesic effect than lumbar epidural injection 3 months after the procedure. [21] In this study, the groups’ meaningful pain relief ratios are between 65.4% and 88.3% at the first month and between 50% and 56% at the third month.

Although there are no data on the relationship between the degree of foraminal stenosis and the results of DRG‐PRF treatment, few studies have examined the diameter of the DRG and PRF treatment. Tortora et al. [22] reported a significant decrease in DRG thickness following DRG‐PRF treatment. They found a positive correlation between the change in DRG diameter and pain relief. Therefore, whether an increased stenosis grade would cause volumetric or structural modifications is unclear. It is known that electrical impulses occur within the DRG, and the activity and mechanical sensitivity within the DRG cause pain [9]. So, it may be thought that an increased grade of LFSS with increased degenerative changes may lead to mechanical stimuli of DRG and cause pain. Therefore, no data are available to support this claim.

In the present study, patients were followed up for 3 months, and no long‐term data are available. Park et al. [21] reported in their review, including 10 RCTs and 613 patients, that the effect of DRG‐PRF therapy persists at 3 months, but there is insufficient evidence regarding its impact on function. Leoni et al. [19] found that DRG‐PRF was effective in improving pain and function up to the third month, regardless of coadministered transforaminal epidural injections, in a study that included 252 patients. De et al. [23] reported that DRG‐PRF treatment was effective for pain relief and functional improvement for up to 6 months in their randomized trial, compared with epidural injection alone. Although these trials were performed, the level of evidence regarding the long‐term duration of pain relief with DRG‐PRF is unclear.

The relatively short follow‐up period, the nonevaluation of DRG volume using imaging modalities, the lack of data about equivalent dose of analgesics, and potential selection bias from excluding patients with prior interventions are limitations of this study.

## 5. Conclusion

DRG‐PRF treatment is an effective modality for improving pain and function in patients with LFSS for all radiological grades of stenosis, with higher NRS and DN4 scores in Grade 3 patients. Studies with larger sample sizes and extended follow‐up periods will provide more detailed information about this issue.

## Funding

No financial support was received for the study.

## Conflicts of Interest

The authors declare no conflicts of interest.

## Data Availability

The data that support the findings of this study are available on request from the corresponding author. The data are not publicly available due to privacy or ethical restrictions.
